# Routine surveillance of asymptomatic healthcare personnel for severe acute respiratory coronavirus virus 2 (SARS-CoV-2): Not a prevention strategy

**DOI:** 10.1017/ice.2020.1428

**Published:** 2021-01-11

**Authors:** Erica S. Shenoy, David J. Weber

**Affiliations:** 1 Infection Control Unit, Massachusetts General Hospital, Boston, Massachusetts; 2 Division of Infectious Diseases, Massachusetts General Hospital, Boston, Massachusetts; 3 Harvard Medical School, Boston, Massachusetts; 4 Division of Infectious Disease, School of Medicine, University of North Carolina at Chapel Hill, Chapel Hill, North Carolina; 5 Department of Hospital Epidemiology, UNC Medical Center, Chapel Hill, North Carolina

As capacity for severe acute respiratory coronavirus virus 2 (SARS-CoV-2) diagnostics has expanded, both with assay types (nucleic acid amplification tests, NAATs, antigen tests, and serology) and specimen collection options (nasopharyngeal, NP; oropharyngeal, OP; saliva; mid-turbinate, MT; anterior nares, AN), interest in the use of routine, serial screening of asymptomatic individuals in a variety of settings has expanded. Notably, the use of asymptomatic surveillance in higher education^1^ and professional^2^ and nonprofessional athletics^3^ has become commonplace, but transmission in these settings has also been linked to lapses in implementation of basic infection prevention practices such as masking and physical distancing.^4–6^ Given the considerable interest in asymptomatic surveillance in areas outside of healthcare, the question of the utility of routine screening among healthcare personnel (HCP) in acute-care facilities has been raised.

In this focused review, we describe the reported risk of acquisition of infection after HCP exposures to occultly infected patients, the risk acquisition of infection by patients exposed to occultly infected HCP, and the prevalence of asymptomatic infection among HCP in settings where screening has been implemented. We also assess the potential role or routine surveillance of asymptomatic HCP to reduce the risk of nosocomial transmission from HCP-to-HCP and HCP-to-patient. We report on the early experience of acute-care facilities that have offered screening of asymptomatic HCP outside confirmed exposures, and we conclude with considerations for facilities considering offering screening, either “on demand” or as part of routine surveillance.

## Risk of HCP infection after exposure to occultly infected patients

Multiple infection prevention measures in healthcare facilities have been widely implemented, including universal masking of HCP, patients, and visitors, screening for symptoms and exposures and appropriate isolation of patients and visitors, testing of symptomatic patients as well as targeted testing of asymptomatic patients (ie, after known exposures, prior to or upon admission to a healthcare facility, and prior to specific high-risk procedures) as well as appropriate isolation and use of personal protective equipment (PPE) by HCP for patients with suspected or confirmed COVID-19.^7,8^ In this setting, the risk of transmission from occultly infected patients appears to be low. This assessment is based on several published investigations of exposures to HCP (Table 1) demonstrating association between universal masking and decreasing incidence of infection.^9^ In addition, seroprevalence studies have generally failed to demonstrate an association between caring for patients with suspected or known COVID-19 and HCP infections, but they have shown relationships between household contacts^10^ and lack of universal mask use when caring for patients.^11^ Several healthcare facility clusters of HCP infection, however, have been linked to HCP-to-HCP transmission tied to eating, drinking, carpooling, and other social events during which infection prevention measures were not followed.^12–14^

**Table 1.** Risk of Infection After HCP Exposure to Occultly Infected Patients

Publication	Date, Country	Brief Description of Occultly Infected Patient and Exposure	Details Regarding PPE	HCP Exposed, Level of Risk of Exposure	No. of Subsequent Infections	Details/Limitations	Rate
Ng et al,^19^ *Ann Intern Med* 2020	February 2020, Singapore	Patient with occult COVID-19 admitted to hospital; developed respiratory distress on HD 2, intubated by emergency airway team; difficult intubation requiring use of video laryngoscope and airway bougie; mechanical ventilation ×3 d; NP positive for SARS-CoV-2 upon extubation	35 HCP wore surgical masks; 6 wore N95 respirators	41 HCP with exposure to AGP for at least 10 min <2 m from patient.	0	All HCP isolated for 2 weeks during which they had daily symptom monitoring, twice daily temperature measurements; NP swabs processed by PCR on first day of home isolation (day 1, 2, 4, or 5 after last exposure) and on day 14	0.0%
Burke et al,^20^ *Morbid Mortal Wkly Rep* 2020	February 2020, United States	Contact tracing of 12 patients with travel-related COVID-19, including 222 HCP with close contact^b^	Not described.	222	0	Active symptom monitoring during exposure window; only symptomatic exposed individuals were tested for SARS-CoV-2 by PCR. The numbers of HCP who developed symptoms and were tested are not specified. Threshold for testing in HCP might have been lower than for other exposed individuals	0.0%
Heinzerling et al,^21^ *Morbid Mortal Wkly Rep* 2020^a^	February 2020, United States	Patient managed on standard precautions for 4 days during which the patient underwent multiple AGPs, including nebulizer treatments, bilevel positive airway pressure, endotracheal intubation, and bronchoscopy; identified as SARS-CoV-2 after transfer to another facility (see Bays et al^22^ for exposure investigation of this patient at the second hospital)	HCP stratified as high, medium, and low risk per CDC; risk stratification provided for 43 who developed symptoms and were tested: high (n=5), medium (n=36), and low (n=2). Among 3 diagnosed with COVID-19, 2 had high risk (frequent close contact during BiPAP, intubation with no facemask, respirator, gown or gloves) and 1 had medium risk exposures (close contact for 2 h wearing a face mask inconsistently; wearing gloves, no eye protection)	121	3	Active symptom monitoring during the exposure window; only symptomatic exposed individuals were tested for SARS-CoV-2 by PCR.	2.5%
Bays et al,^22^ *Infect Control Hosp Epidemiol* 2020^a^	February and March 2020, United States	Describes exposure investigation related to 2 occultly infected patients. Patient 1 was transferred on from a community hospital (community hospital exposure is described in Heinzerling et al^21^) to hospital B. Patient 2 was transferred from another community hospital to hospital B and was on standard precautions for 14 days prior to suspicion for COVID-19 during which the patient was intubated and had bronchoscopy performed.	Patient 1 exposures included high (n=15), medium (n=73), and low (n=59) risk. Patient 2 exposures included high (n=20), medium (n=59), and low (n=66) risk.	147 145	0 5 confirmed 2 possible	Active symptom monitoring during the exposure window; only symptomatic exposed HCP were tested for SARS-CoV-2 by PCR. Active symptom monitoring during the exposure window; symptomatic and a subset of asymptomatic exposed HCP were tested for SARS-CoV-2 by PCR. Of 5 confirmed cases, 4 were present for intubation without adequate PPE, the fifth had direct contact for several days without PPE and during a break in the vent circuit. Two possible cases were among staff who had direct patient contact during AGPs without adequate PPE.	2.4%
Ghinai et al,^23^ *Lancet* 2020^c^	February 2020, United States	Person-to-person spread in household between 2 patients and report of exposures from those two patients within community and healthcare setting	Not described however, healthcare exposures from patient 2 are noted in non-hospitalized settings because the patient was appropriately isolated upon admission.	75	0	Active symptom monitoring during the exposure window; symptomatic exposed individuals were tested for SARS-CoV-2 by PCR; a subset of asymptomatic HCP were tested.	0.0%
Cheng et al,^24^ *JAMA Intern Med* 2020	January–March 2020, Taiwan	Prospective case study of confirmed COVID-19 patients and their close contacts; 698 close contacts were identified in healthcare settings.	Close contact defined as contacting the index case within 2 m without appropriate PPE; no minimum time requirement. Appropriate PPE depended on the exposure setting; during AGPs, N95 required.	698	6	Active symptom monitoring during the exposure window; symptomatic exposed individuals were tested for SARS-CoV-2 by PCR; asymptomatic HCP were also tested as they were considered high-risk population. Repeat testing of asymptomatics only conducted if symptoms developed during the exposure period.	0.9%
Baker et al,^25^ *Infect Control Hosp Epidemiol* 2020	March 2020, United States	Patient admitted to hospital and on Standard Precautions through HD 13 at which point he developed acute respiratory failure; determination made that he was likely infected at the time of admission. The patient was not wearing a mask; on HD7, a new universal masking policy went into effect and HCP work surgical masks.	Close contacts defined as ≥ 10 cumularive minutes of face-to-face contact within 2 m. Median cumulative time with patient was 45 m (range, 10–720 min)	43	2	Active symptom monitoring; all exposed HCP were offered testing for SARS-CoV-2 by PCR, regardless of symptoms. 8 of 44 developed symtpoms and 3 tested positive. Of 36 asymptomatic HCP, 29 were tested and all negative. One HCP who was identified as infected was determined to have had a household exposure and thus was removed from the denominator and numerator for calculations.	4.7%
						**Average**	**1.2%**

Note. HD, hospital day; NP, nasopharyngeal; PCR, polymerase chain reaction; HCP, healthcare personnel; AGP, aerosol-generating procedure.

a Hospital A in Heinzerling et al^21^ is described in detail in Bays et al,^22^ where hospital B is also described. Data presented for Heinzerling include only those from hospital A. Data included from Bays et al pertains to hospital B contact tracing investigation (investigation 1A and 2).

b Close contact defined by CDC at the time: “Examples of close contact with a patient or with infectious material could include spending prolonged time within 6 feet of the patient, conducting or being present during an aerosol-generating procedure, or direct contact with the patient’s secretions or excretions.”

c Exposures related to patient 2 are included in this table because Patient 1 was described in Burke et al; 75 unique HCP contacts are included (personal communication from R Burke to E Shenoy, August 19, 2020).

## Risk of patient infection after exposure to occultly infected HCP

At least 1 study has systematically approached the risk to exposed patients from occultly infected HCP, estimated at 0.4%. Baker et al^15^ identifed exposed patients between March and June 2020. After the study had begun, based on changes in public health guidance, all exposed patients were referred for testing regardless of symptom status. During this time, 238 exposed patients were identified, some with >1 exposure, for 253 exposures by 60 HCP. In 87 exposures, neither patient nor HCP were wearing face masks; in 166 exposures, only the HCP was wearing a face mask. Testing for SARS-CoV-2 by PCR was performed in 92 of 253 exposures, of which 2 resulted positive. The first exposure included unmasked face-to-face interaction for 30 minutes in the outpatient setting, and the second patient was unmasked for 10 minutes with a masked infected HCP, but this patient was also identified as the close contact of a household case, and the infection was attributed to the household.

## Prevalence of asymptomatic infection among HCP

Some academic health centers have offered testing to asymptomatic HCPs without known exposures (ie, for indications other than those recommended at this time). We are not aware at this time of any such practices that are mandatory, or that require repeated testing. A limited review of existing programs and results are provided (Table 2). The overall prevalence among this population is uniformly low and approximates that of institutes of higher education that have implemented routine serial screening. The Massachusetts Department of Public Health, which tracks the 7-day weighted average of tests by molecular methods, notes a recent positive rate of 0.3%.^16^

**Table 2.** Reported Prevalence of SARS-CoV-2 Infection Among Asymptomatic HCP

Location, Start Date	Brief description	Total Tests Performed	No. of Cases Detected	Rate	Type of Test	Reference
Brigham and Women’s Hospital, 9/25/2020^a^	Testing of asymptomatic employees was initiated as part of a hospital cluster, though offered broadly to all employees on campus.	10,840 in 7,999 HCP	14	0.2%	Dry AN swab, self-collected, processed by PCR	26
Massachusetts General Hospital, 10/27/2020	Asymptomatic employees without known exposure were offered voluntary testing, free of charge; limit 1 per week.	5,081 HCP	21	0.4%	Dry AN swab, self-collected, processed by PCR	27
National Institutes of Health patient care providers, 5/21/2020; NIH campus, 8/11/2020	Asymptomatic testing was voluntary but highly encouraged; clinical staff are encouraged to test weekly. Pooled specimen approach was used.	38,450 in 8,578 HCP	33	0.4%	Initially NP swab; beginning 9/14/2020 saliva; mid-turbinate also accepted; processed by PCR	28
University of California–San Francisco, 7/2020	Voluntary testing of asymptomatic employees, trainees, and students, randomly selected, was offered in addition to asymptomatic testing for new and returning trainees and students, new campus housing tenants, childcare staff working in UCSF’s childcare centers, and others.	16,702 in 7,627 HCP	^b^	0.21%	Self-administered, observed, AN	29, 30
Yale New Haven Health System, June–July 2020	Voluntary testing of asymptomatic HCP	11,000	28	0.25%	Details not available	31

Note. HCP, healthcare personnel; AN, anterior nares; NP, nasopharyngeal; NIH, National Institutes of Health; UCSF, University of California–San Francisco.

a Screening was initiated in the setting of a cluster of infections though vast majority of testing was performed in nonexposed HCP. Total infections presented are those that were not attributed to the cluster, and these 14 were removed from the denominator for calculation of proportion positive.

b Data provided did not allow identification of asymptomatic denominator.

## Potential benefits of asymptomatic HCP screening

Testing of asymptomatic HCP will identify some infections that will otherwise go undetected due to lack of prompts for evaluation. The impact of identifying those cases on nosocomial infection is not clear. Although asymptomatic individuals do transmit infection, available literature suggests that the secondary attack rate from asymptomatic individuals is less than for those with symptoms.^17^ More importantly, in the healthcare setting when adherence to infection prevention protocols are in place, the risk of transmission to patients and other HCP appears low. The effect of identifying occultly infected HCP on reduced transmission in the community or household setting is likely higher because of the types of interactions in households, and household settings have been shown to have the highest rates of secondary transmission.^18^ The HCP infection risk is likely higher in community and household settings than in healthcare settings; thus, the identification of asymptomatic HCP may have its greatest effect in limiting transmission in the household setting.

Outside a potential impact on reducing transmission, there may be noninfection prevention benefits to offering HCP testing, including HCP satisfaction through ease of access and some measure of reassurance. This reassurance of a negative test, however, is short-lived and runs a risk of reducing compliance with necessary infection control procedures.

## Potential disadvantages of asymptomatic screening

Will HCP who test negative for SARS-CoV-2 modify their behaviors in a way that could increase risk of transmission, by engaging in more risky behaviors, such as eating or drinking in close proximity with nonhousehold members? Although we are not aware of evidence to support this change in behavior during the current pandemic, observations of lack of compliance with eye protection in our own institutions in settings in which inpatients are all tested for SARS-CoV-2 on admission suggest that HCP are assessing risk of transmission from patients and altering their behavior accordingly (ie, not wearing eye protection when the patient tested negative despite the existing policy to wear eye protection universally).

Even in such a low-prevelance population, the risk of false-positive results, which has generally been very low in nucleic acid amplification tests (NAATs) but higher with some antigen tests, must also be considered. Facilities will need to decide in advance whether all positive results will be considered to be true infections, or whether additional assessment of each case is required to confirm or refute active infection, taking into account the impact on return-to-work status and exposure investigations. We are unaware of data on testing of asymptomatic HCP in which positive tests were confirmed as “true” positives by follow-up serologic tests.

## Practical considerations

Any healthcare facility considering asymptomatic HCP screening either as voluntary or mandatory programs must be aware of practical considerations, such as the frequency of testing, the type of assay, the specimen type, and pooling strategies, all of which can affect the sensitivity of the assay and the timing of detection. Observed self-collection may be an option depending on the specimen type and may introduce efficiencies in testing cohorts of HCP at the same time, with appropriate infection prevention protocols in place. Unobserved self-collection should be undertaken with caution given the possibility of poor sample collection and false-negative results. In low-prevalence populations, false-positive results may be a concern, and facilities may consider protocols to follow-up positive screening tests with confirmatory or other tests. Facilities may consider whether to offer testing to all HCP or specific groups; however, caution should be taken when focusing on those HCP considered at “higher risk of infection” due to direct patient care because the most likely source of infection in all HCP is community exposure. Thus focusing on HCP with higher risk of unrecognized community exposures may be considered. Some facilities may alternatively undertake surveillance among HCP in whom infection would pose a greater risk to patients based on the types of interactions or patient populations with whom they interact. This strategy should also be considered with caution because the risk to exposed patients when infection prevention measures are in place (ie, universal masking of HCP, daily symptom monitoring, and masking of patients whenever possible) is low.

In addition to the cost of establishing and maintaining a testing program, the additional resources that will be required for contact tracing to identify potential exposures to other HCP or patients due to lapses in infection prevention protocols must be considered. These include staffing and other support from infection prevention programs and occupational health staff. The demand for testing may exceed budgeted resources.

In summary, the low risk of nosocomial transmission from patient to HCP and from HCP to patient, as well as the low prevalence of asymptomatic SARS-CoV-2 infection among HCP suggests that current infection prevention measures in place are effective. The addition of routine asymptomatic surveillance to decrease transmission in healthcare facilities should not be pursued as a primary infection prevention strategy, and institutions that consider offering such screening will need to consider the many practical implications. With increasing community prevalence across much of the United States, reinforcing the known, effective infection prevention strategies is of paramount importance. Healthcare does not operate in a bubble and routine screening of asymptomatic HCP will not create one.

## References

[1] Testing, screening, and outbreak response for institutions of higher education (IHEs). Centers for Disease Control and Prevention website. https://www.cdc.gov/coronavirus/2019-ncov/community/colleges-universities/ihe-testing.html. Published 2020. Accessed January 8, 2021.

[2] DiFiori JP , Green G , Meeuwisse W , Putukian M , Solomon GS , Sills A. Return to sport for North American professional sport leagues in the context of COVID-19. Br J Sports Med 2020. doi: 10.1136/bjsports-2020-103227. 32967854

[3] Resocialization of collegiate sport: developing standards for practice and competition 2020. National Collegiate Athletic Association website. http://www.ncaa.org/sport-science-institute/resocialization-collegiate-sport-developing-standards-practice-and-competition. Published November 17, 2020. Accessed January 8, 2021.

[4] Murray MT , Riggs MA , Engelthaler DM , et al. Mitigating a COVID-19 outbreak among major league baseball players—United States, 2020. Morb Mortal Wkly Rep 2020;69:1542–1546. 10.15585/mmwr.mm6942a4 PMC7583504 33090983

[5] Teran RA , Ghinai I , Gretsch S , et al. COVID-19 outbreak among a university’s men’s and women’s soccer teams—Chicago, Illinois, July–August 2020. Morb Mortal Wkly Rep 2020;69:1591–1594. 10.15585/mmwr.mm6943e5 PMC7659918 34463672

[6] Szablewski CM , Chang KT , Brown MM , et al. SARS-CoV-2 transmission and infection among attendees of an overnight camp—Georgia, June 2020. Morb Mortal Wkly Rep 2020;69:1023–1025. 10.15585/mmwr.mm6931e1 PMC7454898 32759921

[7] Interim infection prevention and control recommendations for healthcare personnel during the coronavirus disease 2019 (COVID-19) pandemic. Centers for Disease Control and Prevention website. https://www.cdc.gov/coronavirus/2019-ncov/hcp/infection-control-recommendations.html. Published 2020. Accessed January 8, 2021.

[8] Healthcare facilities: managing operations during the COVID-19 pandemic. Centers for Disease Control and Prevention website. https://www.cdc.gov/coronavirus/2019-ncov/hcp/guidance-hcf.html. Published 2020. Accessed January 8, 2021.

[9] Wang X , Ferro EG , Zhou G , Hashimoto D , Bhatt DL. Association between universal masking in a health care system and SARS-CoV-2 positivity among healthcare workers. JAMA 2020;324:703–704. 10.1001/jama.2020.12897 PMC7362190 32663246

[10] Steensels D , Oris E , Coninx L , et al. Hospital-wide SARS-CoV-2 antibody screening in 3056 staff in a tertiary center in Belgium. JAMA 2020;324:195–197. 32539107 10.1001/jama.2020.11160 PMC7296458

[11] Self WH , Tenforde MW , Stubblefield WB , et al. Seroprevalence of SARS-CoV-2 among frontline healthcare personnel in a multistate hospital network—13 academic medical centers, April–June 2020. Morb Mortal Wkly Rep 2020;69:1221–1226. 10.15585/mmwr.mm6935e2 PMC7470460 32881855

[12] Ellsworth M , Chang M , Ostrosky-Zeichner L. Mind the gap: the hospital breakroom. Am J Infect Control 2020;48:1285. 32562713 10.1016/j.ajic.2020.06.179 PMC7832819

[13] Çelebi G , Pişkin N , Çelik Bekleviç A , et al. Specific risk factors for SARS-CoV-2 transmission among healthcare workers in a university hospital. Am J Infect Control 2020;48:1225–1230. 32771498 10.1016/j.ajic.2020.07.039 PMC7409872

[14] Haessler S. Anatomy of a COVID-19 Outbreak. SHEA Town Hall #17, August 2, 2020. https://www.facebook.com/watch/232181883513788/795543604539707/.

[15] Baker MA , Fiumara K , Rhee C , et al. Low risk of coronavirus disease 2019 (COVID-19) among patients exposed to infected healthcare workers. Clin Infect Dis 2020. doi: 10.1093/cid/ciaa1269. PMC7499548 32856692

[16] Dashboard of Public Health Indicators—November 17, 2020. Massachusetts Department of Public Health website. https://www.mass.gov/doc/covid-19-dashboard-november-17-2020/download. Accessed January 8, 2021.

[17] Luo L , Liu D , Liao X , et al. Contact settings and risk for transmission in 3,410 close contacts of patients with COVID-19 in Guangzhou, China: a prospective cohort study. Ann Intern Med 2020. doi: 10.7326/M20-2671. PMC7506769 32790510

[18] Grijalva CG , Rolfes MA , Zhu Y , et al. Transmission of SARS-COV-2 infections in households—Tennessee and Wisconsin, April–September 2020. Morb Mortal Wkly Rep 2020;69:1631–1634. 10.15585/mmwr.mm6944e1 PMC7643897 33151916

[19] Ng K , Poon BH , Kiat Puar TH , et al. COVID-19 and the risk to healthcare workers: a case report. Ann Intern Med 2020;172:766–767. 32176257 10.7326/L20-0175 PMC7081171

[20] Burke RM , Midgley CM , Dratch A , et al. Active monitoring of persons exposed to patients with confirmed COVID-19—United States, January–February 2020. Morb Mortal Wkly Rep 2020;69:245–246. 10.15585/mmwr.mm6909e1 PMC7367094 32134909

[21] Heinzerling A , Stuckey MJ , Scheuer T , et al. Transmission of COVID-19 to healthcare personnel during exposures to a hospitalized patient—Solano County, California, February 2020. Morb Mortal Wkly Rep 2020;69:472–476. 10.15585/mmwr.mm6915e5 PMC7755059 32298249

[22] Bays DJ , Nguyen MH , Cohen SH , et al. Investigation of nosocomial SARS-CoV-2 transmission from two patients to healthcare workers identifies close contact but not airborne transmission events. Infect Control Hosp Epidemiol 2020. doi: 10.1017/ice.2020.321. PMC8387689 32618530

[23] Ghinai I , McPherson TD , Hunter JC , et al. First known person-to-person transmission of severe acute respiratory syndrome coronavirus 2 (SARS-CoV-2) in the USA. Lancet 2020;395:1137–1144. 32178768 10.1016/S0140-6736(20)30607-3 PMC7158585

[24] Cheng HY , Jian SW , Liu DP , et al. Contact tracing assessment of COVID-19 transmission dynamics in taiwan and risk at different exposure periods before and after symptom onset. JAMA Intern Med 2020;180:1156–1163. 32356867 10.1001/jamainternmed.2020.2020 PMC7195694

[25] Baker MA , Rhee C , Fiumara K , et al. COVID-19 infections among HCWs exposed to a patient with a delayed diagnosis of COVID-19. Infect Control Hosp Epidemiol 2020;41:1075–1076. 32456720 10.1017/ice.2020.256 PMC7276498

[26] Brigham Health. Statement for the Media Regarding COVID-19 Cluster. October 16, 2020. https://www.brighamandwomens.org/about-bwh/newsroom/press-releases-detail?id=3684.

[27] Massachusetts General Hospital. Mass General Minute: Behind the Curve. Internal Communication, November 17, 2020.

[28] Henderson DK. SARS-CoV-2 Testing at the NIH. SHEA Town Hall #30, November 15, 2020. https://www.facebook.com/watch/232181883513788/795543604539707/.

[29] Office of the Chancellor. COVID-19 asymptomatic testing program for onsite employees, students, and trainees. University of California–San Francisco website. https://chancellor.ucsf.edu/blog/covid-19-asymptomatic-testing-program. Published 2020. Accessed January 8, 2021.

[30] UCSF COVID-19 dashboard: University of California–San Francisco website. https://coronavirus.ucsf.edu/dashboard. Published 2020. Accessed January 8, 2021.

[31] Successful employee testing program yields reassuring results. Yale New Haven Health website. https://www.ynhhs.org/publications/bulletin/071620/successful-employee-testing-program-yields-reassuring-results.aspx. Published 2020. Accessed January 8, 2021.

